# A novel approach for multi-SNP GWAS and its application in Alzheimer’s disease

**DOI:** 10.1186/s12859-016-1093-7

**Published:** 2016-07-25

**Authors:** Paul M. Bodily, M. Stanley Fujimoto, Justin T. Page, Mark J. Clement, Mark T. W. Ebbert, Perry G. Ridge, the Alzheimer’s Disease Neuroimaging Initiative

**Affiliations:** 1 Computer Science Department, Brigham Young University, Provo, 84602-6576 UT USA; 2 Department of Biology, Brigham Young University, Provo, 84602-6576 UT USA

**Keywords:** GWAS, Multi-SNP GWAS, Alzheimer’s disease, Epistasis

## Abstract

**Background:**

Genome-wide association studies (GWAS) have effectively identified genetic factors for many diseases. Many diseases, including Alzheimer’s disease (AD), have epistatic causes, requiring more sophisticated analyses to identify groups of variants which together affect phenotype.

**Results:**

Based on the GWAS statistical model, we developed a multi-SNP GWAS analysis to identify pairs of variants whose common occurrence signaled the Alzheimer’s disease phenotype.

**Conclusions:**

Despite not having sufficient data to demonstrate significance, our preliminary experimentation identified a high correlation between GRIA3 and HLA-DRB5 (an AD gene). GRIA3 has not been previously reported in association with AD, but is known to play a role in learning and memory.

**Electronic supplementary material:**

The online version of this article (doi:10.1186/s12859-016-1093-7) contains supplementary material, which is available to authorized users.

## Background

Until recently linkage studies were the best approach to identify genes responsible for genetic diseases. However, linkage studies are most successful in monogenic disorders with highly penetrant variants, and were most often only possible in families. Unfortunately, the majority of genetic diseases are complex and therefore their genetic architectures could not be studied with linkage studies. Following completion of the human genome and the development of accurate SNP arrays, the Human HapMap project [1] cataloged the majority of common variants in the human genome. This catalog of SNPs facilitated the creation of genome-wide association studies (GWAS) [2]. In a GWAS, the co-occurence of a given SNP and a phenotype are assessed. SNPs present (or absent) significantly more often in individuals with a particular phenotype or more extreme phenotype, are reported as disease markers (i.e., genomic variation correlated with a trait, but not necessarily causative). GWAS can be used to study both quantitative and binary phenotypes, and were the first effective approach for studying the genetics of complex traits.

In 2005, Haines et al. [3] conducted the first GWAS, examining statistical significance between single SNPs and age-related macular degeneration. In the decade since, the success of this technique has been used to identify genetic factors associated with dozens of traits (e.g., coronary heart disease, type-1 diabetes, type-2 diabetes, rheumatoid arthritis, Crohn’s disease, bipolar disorder, hypertension, Alzheimer’s disease, and others [4]). The GWAS catalog co-curated by the National Human Genome Research Institute and the European Bioinformatics Institute contains reported associations for thousands of GWAS [5].

Unfortunately, GWAS have several limitations. First, most GWAS markers are thought to be non-functional, so while the marker may provide insights into regions of the genome important to a particular phenotype, unless the marker is functional it typically does not provide information about the specific biological mechanisms driving disease. Second, the majority of GWAS markers have only a very modest effect on risk for disease. Third, despite thousands of GWAS performed using progressively bigger datasets, collectively GWAS SNPs only explain a portion—often a small portion—of the total estimated genetic variance for a given trait [6].

A number of explanations exist for the unexplained genetic variance. One possibility is that gene-gene (i.e., epistasis) interactions are a feature of the genetic architecture of these traits. GWAS assume an additive model (i.e., SNPs confer disease risk independent of other SNPs) and therefore cannot be used to detect epistatic interactions. Numerous approaches have been attempted to identify epistasis including multifactor dimensionality reduction, regression (i.e., GWAS with interaction terms), and others [7], each with different pros and cons. In this manuscript we present a novel multi-SNP GWAS approach for identifying epistatic interactions and demonstrate its utility in Alzheimer’s disease (AD).

AD is the most common cause-of-death with no effective treatments and has a rapidly increasing incidence worldwide [8]. Additionally, AD is the ideal phenotype to use to demonstrate the utility of our approach for two reasons: 1) epistasis has a role in the genetic architecture of AD [7, 9, 10], and 2) despite very large GWAS (Table 1) and the identification of several rare SNPs [11–13], a substantial portion of the genetic variance remains unexplained [14].

**Table 1:** Genes most highly associated with Alzheimer’s disease

Gene	dbSNP ID	Chr:Pos
APOE e2	rs7412	19:45412078
APOE e4	rs429358	19:45411940
CR1	rs6656401	1:207692048
BIN1	rs6733839	2:127892809
CD2AP	rs10948363	6:47487761
EPHA1	rs11771145	7:143110761
CLU	rs9331896	8:27467685
MS4A6A	rs983392	11:59923507
MS4A4E	rs670139	11:59971794
PICALM	rs10792832	11:85867874
ABCA7	rs4147929	19:1063442
CD33	rs3865444	19:51727961
HLA-DRB5/HLA-DRB1	rs9271192	6:32578529
PTK2B	rs28834970	8:27195120
SORL1	rs11218343	11:121435586
SLC24A4/RIN3	rs10498633	14:92926951
DSG2	rs8093731	18:29088957
INPP5D	rs35349669	2:234068475
MEF2C	rs190982	5:88223419
NME8	rs2718058	7:37841533
ZCWPW1	rs1476679	7:100004445
CELF1	rs10838725	11:47557870
FERMT2	rs17125944	14:53400628
CASS4	rs7274581	20:55018259

We only included SNPs that are consistently replicated in AD GWAS studies (Lambert et al. [17] and http://www.AlzGene.org)

## Methods

### Dataset

This research used 802 whole genomes (279 control, 191 case, 332 unknown, and 444 males and 358 females) from the Alzheimer’s Disease Neuroimaging Intiative (ADNI). The genomes were processed by ADNI using the Burrows-Wheeler aligner (BWA) [15] and the best practices of the Genome Analysis Toolkit (GATK) [16]. Genomes were obtained from the ADNI database (adni.loni.usc.edu). The ADNI was launched in 2003 as a public-private partnership, led by Principal Investigator Michael W. Weiner, MD. The primary goal of ADNI has been to test whether serial magnetic resonance imaging (MRI), positron emission tomography (PET), other biological markers, and clinical and neuropsychological assessment can be combined to measure the progression of mild cognitive impairment (MCI) and early AD. For up-to-date information, see www.adni-info.org.

### Single-SNP GWAS analysis

Single-SNP GWAS uses a modified form of linkage disequilibrium (LD) to infer relationships between single SNPs and observed phenotypes. In order to understand this approach, it is necessary to understand LD and how it is usually applied in genetic analyses. LD is a measure of how often two genomic features are inherited together within a population of interest, compared to how often they “should” be inherited together (i.e., the difference between the observed co-occurence of two SNPs and the expected co-occurence of the two SNPs). LD *D*, the co-occurrence of events (SNPs) *A*_1_ and *B*_1_ (as opposed to *A*_2_ and *B*_2_, respectively) is calculated as:

$$D = p(A_{1} \land B_{1}) - p(A_{1}) \ast p(B_{1}) $$

In the simple case often seen in genetics, these events are different nucleotides (A, C, T, or G) that exist at specific positions in the genome. We would like our LD to reflect the likelihood of observing both SNPs together (e.g., if *A*_1_ occurs then we know confidently that *B*_1_ also occurs, and vice versa). Unfortunately, *D* does not provide this information. However, a second measure of LD, *r*^2^, based on *D*, is a measure of how closely related the two events (SNPs) are. A measure of *r*^2^=1.0 means they provide the exact same information, or always co-occur. *D* is converted to the Pearson correlation coefficient *r* by the following:

$$r = D / \sqrt{p(A_{1}) \ast p(A_{2}) \ast p(B_{1}) \ast p(B_{2})} $$

In GWAS, LD is used to select which SNPs from the genome to analyze. For example, if two SNPs provide the exact same information (i.e., they always or almost always are inherited together), then only one of the SNPs is analyzed. This reduces the total number of tests (i.e., preserves statistical power) by eliminating redundant tests.

In our research, rather than using *p*-values from a regression to assess the relationship between a SNP (or multiple SNPs) and AD case/control status (i.e., which SNPs are correlated with AD), we calculated *r*^2^ between each SNP in the dataset and AD case/control status. In this approach, the two events we are measuring are the co-occurence of a SNP and AD case/control status (i.e., do case status and a particular SNP co-occur with high confidence). We accomplished this by writing our own algorithm that computes LD between each SNP and AD case/control status.

An outline of our single-SNP GWAS algorithm is given in Algorithm 1. Note that there are multiple genotype values of *A*_*i*_ because there are two haplotypes. The computation of Pearson’s *r* is as described above given the computed probabilities. This approach on a set of individuals, *S*, and a set of SNPs, *L*, runs in *O*(∥*S*∥×∥*L*∥) time and with *O*(1) space.



### Multi-SNP GWAS analysis

To extend to multi-SNP GWAS, we calculate LD between two SNPs and a phenotype. Comparing the co-occurence of two SNPs and a trait results in eight different calculations for *D* of the form:

$$D_{i,\,j,\,k} = p(A_{i} \land B_{j} \land C_{k}) - p(A_{i}B_{j}) \ast p(C_{k}) $$

Unlike in the single-SNP case, the magnitude of *D* may differ between each of the eight comparisons. Because we are concerned with the possibility of a specific combination of alleles impacting the trait, we take the maximum among all eight values for *D*. Next we calculate *r*^2^ using the specific combination of alleles as one event and any other combination (three possibilities, in the two SNP case) as the alternate outcome for that event:

$$d_{i,\,j,\,k} = \sqrt{p(A_{i}B_{j}) * (1-p(A_{i}B_{j})) * p(C_{1}) * p(C_{2})} $$

This results in the following equation for *r*:

$$r = {max}_{i,\,j,\,k} (D_{i,\,j,\,k} / d_{i,\,j,\,k}) $$

To calculate the correlation of all SNPs with every other SNP would have exceeded our computational resources. We therefore calculated correlations between all SNPs and a subset of the SNPs that were located in genes with strongest and most consistent associations with AD (Table 1). Even this matrix was too large to fit into memory, so we computed subsections of the matrix in parallel on different machines. We found 2101 pairs of SNPs with an *r*^2^ correlation greater than 0.04. These we plotted using R(Studio) to look for genes that had a (relatively) high correlation with other SNPs, as the relationship between two genes could provide important insights into disease processes.



An outline of our multi-SNP parallelized GWAS algorithm is given in Algorithm 2. The required *START* and *END* allow the task to be partitioned and run in parallel. There are multiple genotype values of *A*_*i*_ and *B*_*j*_ because there are two haplotypes. Our solution on on a set of individuals, *S*, and a set of SNPs, *L*, runs in *O*(∥*S*∥×∥*L*∥^2^/*k*) time and with *O*(∥*L*∥) space for each of *k* parallel runs (for our run *k*=201 with each run allocated 32GB of RAM and a wall time of 6 h).

## Results

### Single-SNP GWAS analysis

**Fig. 1:**
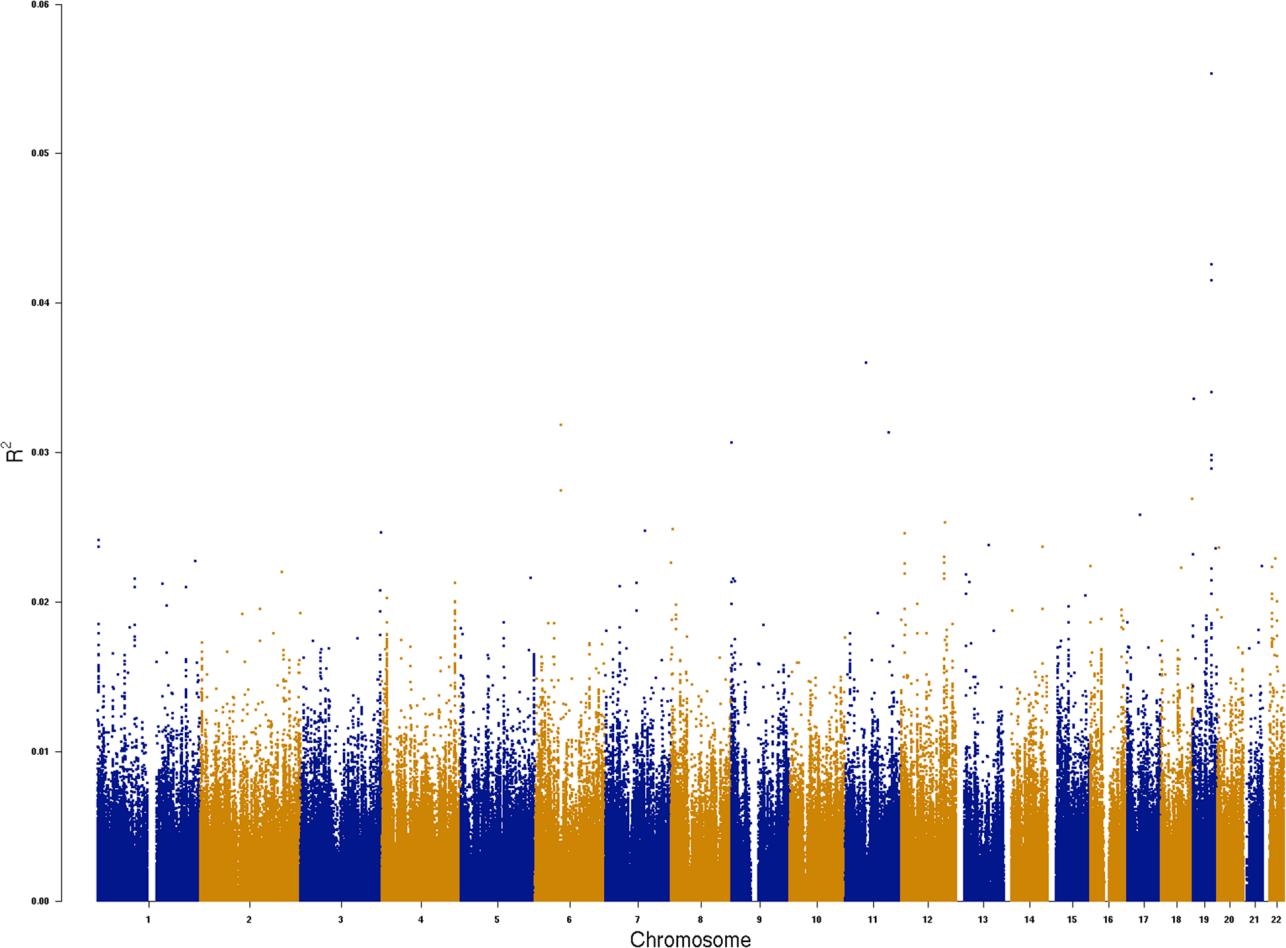
Squared Pearson’s coefficient for all SNPS in gene-coding regions. Note the outlying values in chromosome 19 which correspond to the regions of the APOE_e2/3/4 and PVRL2 gene-coding regions

We knew in advance that our dataset would be insufficient to achieve statistical significance and present our results as a demonstration of how it could be used in a larger dataset. Although Manhattan Plots are usually calculated using *p*-values, we used the *r*^2^ values to compare different SNPs (calculated using our algorithm). For each chromosome we computed and plotted the *r*^2^ of each SNP in all gene-coding regions (regardless of previous implication in AD). This analysis provided a comparison to traditional GWAS analyses as well as a comparison to our subsequent multi-SNP analysis (Fig. 1).

**Fig. 2:**
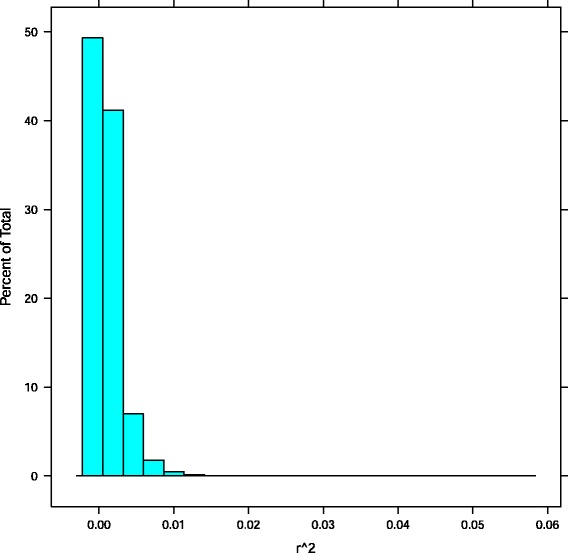
Single-SNP *r* ^2^ distribution. Histogram reflecting the frequency of binned *r* ^2^ values or correlations between single SNPs and AD phenotype

In this demonstration of our approach, we report 29 different SNPs with *r*^2^≥ 0.025 (a very conservative cutoff, which corresponds to a *p*=1.578*e*−5 (Fig. 2)) in 14 genes (Table 2). Of these 14 genes, we found 3 (APOE, TOMM40, and PVRL2) that have been previously implicated in AD.

**Table 2:** SNPs with *r* ^2^≥ 0.025

Gene	Chr:Pos	r^2
KHDRBS2KHDRBS2	6:62728214	0.027466
KHDRBS2	6:62728218	0.0318925
CBWD1	9:136147	0.0306714
RP11-347H15.1	11:50136388	0.0360252
RP11-886D15.1	11:104457155	0.0313399
C12orf23	12:107350959	0.0253392
RP11-220C2.1	17:31004608	0.0258624
NFATC1	18:77243594	0.0269173
ABHD17A	19:1879687	0.0336168
PVRL2	19:45392254	0.0295325
TOMM40	19:45406673	0.0298293
APOE	19:45410002	0.0415463
APOE	19:45411941	0.0553697
APOC1	19:45418790	0.0289349
APOC1	19:45421254	0.0340441
APOC1	19:45422160	0.0425822
RP11-265P11.1	X:39085516	0.0293786
RP11-265P11.1	X:39086096	0.0301775
RP11-265P11.1	X:39086353	0.0288563
RP11-265P11.1	X:39098097	0.031864
GS1-256O22.5	X:142421475	0.0288961
GS1-256O22.5	X:142422021	0.0254872
GS1-256O22.5	X:142426750	0.0275156
GS1-256O22.5	X:142429074	0.0276677
GS1-256O22.5	X:142430401	0.026733
GS1-256O22.5	X:142439000	0.0268576
UBL4A	X:153713451	0.0260031
UBL4A	X:153713787	0.0253136
UBL4A	X:153714030	0.0259109

### Multi-SNP GWAS analysis

**Fig. 3:**
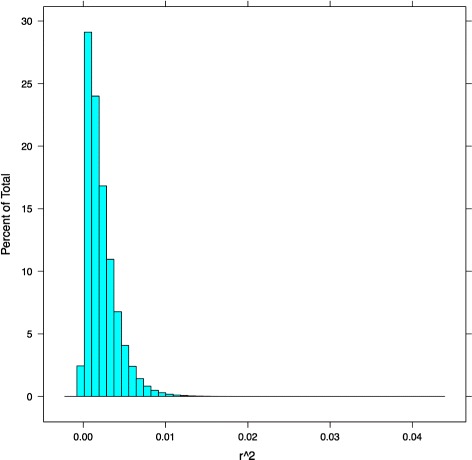
Multi-SNP *r* ^2^ distribution. Histogram reflecting the frequency of binned *r* ^2^ values or correlations between pairs of SNPs and AD phenotype

We calculated correlations between all SNPs and genic SNPs located in genes previously associated with AD (i.e. Table 1), without regard to minor allele frequency (i.e. included both rare and common SNPs). We selected 0.04 as a cutoff for the multi-SNP GWAS analysis (Fig. 3). Of 4 million randomly selected SNPs from the distribution, only 3 had *r*^2^≥ 0.04 (*p*=7.501*e*−7). In practice, a Bonferroni correction could be performed by choosing different *r*^2^ cutoffs based on the corrected alpha. We identified 192 pairs correlated with AD with an *r*^2^≥ 0.04. Additional file 1 lists the AD SNP together with the non-AD SNP and the *r*^2^ correlation with the AD phenotype. The table is sorted by *r*^2^ value (most to least significant). For each pair of genes, only SNP pair with the highest *r*^2^ value was included. The final column indicates how many total significant SNP pairs were found for the given pair of genes. For example, the highest correlation had an *r*^2^ value of 0.0730273 and was found between rs72508453 (in AD gene HLA-DRB5) and a SNP on chromosome 16 at position 6110138 (in non-AD gene RP11-509E10.1). There were 15 significant SNP pairs between these two genes. This could suggest an epistatic relationship between these two genes that is correlated with the AD phenotype.

**Fig. 4:**
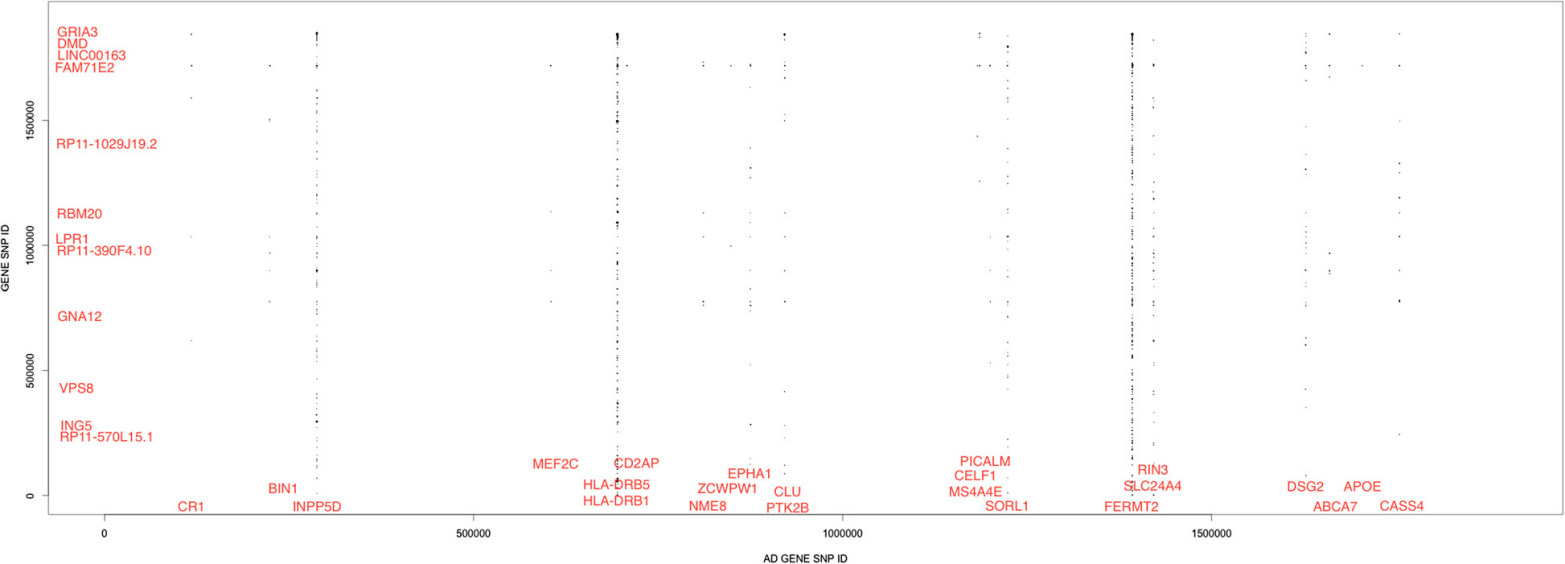
Results of the Multi-SNP GWAS analysis. SNP pairs with *r* ^2^ values >0.04 were plotted. SNP ID is based on SNP order, not SNP position. For genes with several correlated SNP pairs, gene names roughly indicate corresponding SNP IDs (see Additional file 1 for more detailed information). On the X-axis, any AD gene that had correlated SNP pairings is labeled, and on the y-axis any gene that had 44 or more correlated SNP pairings is labeled. To avoid plotting all SNP positions, SNP IDs were used. SNP IDs reflect the same ordering as the associated SNP positions but are limited to include only analyzed, gene-coding SNPs

To visualize our results, we plotted the correlation of AD SNPs (on the X axis) against all genic SNPs (on the Y axis) in Fig. 4. Only SNP pairs with significant *r*^2^ values are shown. Plotting the significant pairs revealed two very clear bands, and an additional three bands of interest. A vertical band indicates that an AD gene has significant epistatic interaction with other non-AD genes. The two clearest bands correspond with SNPs in HLA-DRB5/HLA-DRB1 and FERMT2, the other three bands correspond with SNPs in INPP5D, SORL1, and RIN3/SLC24A4. All but two known AD genes (MS4A6A and CD33) had correlated SNP pairs. 12 different non-AD genes had 44 or more correlated SNP pairings.

Among the 10 SNP pairs with strongest correlation, half of the pairings include a SNP from HLA-DRB5, two from INPP5D, two from FERMT2, and one from PTK2B. All but one of the SNPs from the top 10 pairs mentioned comes from a unique SNP with no prior reported AD involvement. Among the top 10, ELF4 had two SNPs with significant pairings. The first (rs6637686) was correlated with rs78623109 in FERMT2. The second (rs3788847) was correlated with rs377610860 in PTK2B.

## Discussion

Although our sample size is too small to identify statistically significant correlations, this work demonstrates the utility of taking a linkage disequilibrium approach in our single- and multi-SNP GWAS. First, we identified APOE as an important AD gene in the single-SNP GWAS using our new approach. This is hardly novel as two different APOE SNPs, rs7412 and rs429358, are the most established genetic protective/risk factors for AD [8]. However, it is significant because the APOE effect is strong enough that we would expect to detect the signal in even a small dataset. In contrast, all of the other known variations that affect risk for AD are either very rare (PLD3 [11], APP [13], TREM2 [12], etc.), or have very small effect sizes [17]. Consequently, because of our sample size it is not surprising that we did not detect any other Results of the AD markers in the single-SNP GWAS.

Next, we applied our multi-SNP GWAS to identify pairs of SNPs jointly correlated with AD. In principle, epistatic interactions can exist between two genes that individually are not correlated with disease. In this study we tested the pairing of each genic SNP in known AD genes with every other SNP in the dataset. In this analysis there were five strong bands corresponding with AD genes with a large number of identified SNPxSNP pairings.

Among the 10 strongest correlations, ELF4 had two correlated SNPs (one with FERMT2 and one with HLA-DRB5). ELF4 (E74-like factor 4) is a transcription factor involved in immunity and cell cycle control [18–20]. The function of FERMT2 is unknown. However, HLA-DRB5 has a direct role in immunity [21]. From a biological standpoint, an interaction between HLA-DRB5 and ELF4 makes sense, and the immune system has a known role in AD [22].

GRIA3 (glutamate receptor, ionotropc, ampa 3) is another gene with SNPs of potential interest. A SNP in GRIA3 (rs7061304) was the second strongest correlation in our analyses (paired with rs67588672 in HLA-DRB5), and GRIA3 is among the non AD genes with the highest number of correlations. GRIA3 is a glutamate receptor. Glutamate receptors are the primary neurotransmitter receptors in human brains and GRIA3 specifically has a role in learning and memory. Furthermore, GRIA3 has been implicated in numerous disorders in the brain including bipolar disorder, mental retardation, and encephalopathy with epileptic seizures (Rasmussen encephalitis) [23, 24]. Finally, mutations in GRIA3 have been associated with cognitive impairment [25]. Although GRIA3 has not been associated with AD, glutamate receptors have been studied (including members in the same family of genes as GRIA3) for their effect on Alzheimer’s disease based on the hypothesis that malfunction of glutamate receptors leads to AD-specific cell loss [26]. When considering the function of GRIA3, especially in relationship with a known AD SNP (HLA-DRB5), GRIA3 is an attractive candidate gene for further studies.

## Conclusions

In summary, we developed a novel multi-SNP GWAS method and demonstrated its utility in an AD dataset. Using this approach we identified potential epistatic interactions that affect risk for AD. GRIA3, in particular, is especially intriguing and warrants followup studies in larger datasets. Due to the difficulty identifying epistatic interactions, relatively few interactions are known. Future work will focus on developing an appropriate dataset with experimentally validated epistatic interactions for testing new models, integrating known biology of genes in identified interactions to identify mechanisms of synergistic functionality, and modification of the approach to more appropriately ascbribe statistical significance (i.e., *p*-values) to identified interactions.

## Additional file

Additional file 1
**Table 3.** Gene pairs with SNP pairs having *r* ^2^≥ 0.04. (PDF 169 kb)
