# Supplementary material for: A novel approach for multi-SNP GWAS and its application in Alzheimer’s disease
Source: BMC Bioinformatics. 2016 Jul 25;17(Suppl 7):268. doi: 10.1186/s12859-016-1093-7 (PMC4965706; doi:10.1186/s12859-016-1093-7)
Supplement: Additional file 1 — Table 3. Gene pairs with SNP pairs having r 2≥ 0.04. (PDF 169 kb) [file 12859_2016_1093_MOESM1_ESM.pdf]

Table 1 Gene pairs with SNP pairs having $r^2 \geq 0.04$ (pair with highest r^2 value reported)

AD Gene		Other Gene			Total SNP pairs
Gene Name	rs ID or chr:pos	Gene Name	rs ID or chr:pos	r^2	for gene pair
HLA-DRB5	rs72508453	RP11-509E10.1	16:6110138	0.0730273	15
HLA-DRB5	rs67588672	GRIA3	rs7061304	0.0714257	85
HLA-DRB5	rs77149458	WDR63	rs67661067	0.0711551	26
HLA-DRB5	rs1064595	FAM21B	rs201878841	0.0696392	5
INPP5D	2:233994599	SRPK3	rs7052332	0.0678255	6
FERMT2	rs78623109	ELF4	rs6637686	0.0668191	11
FERMT2	rs78623109	FAM71E2	rs36172402	0.0642951	58
PTK2B	rs377610860	ELF4	rs3788847	0.0642159	1
INPP5D	rs10195662	RP11-124B13.1	rs193238269	0.0639601	50
HLA-DRB5	rs116822312	DMD	rs55646021	0.0607229	84
FERMT2	rs77026229	AL590867.1	6:153553842	0.0598909	10
HLA-DRB5	rs187620826	SHROOM2	rs4830419	0.0598423	51
HLA-DRB5	rs140066841	ELF4	rs3788847	0.0593773	7
FERMT2	rs78623109	AC019118.2	rs12714369	0.0591228	15
FERMT2	rs78623109	GYG2	rs5939350	0.0585612	11
FERMT2	rs78623109	RP11-733O18.1	rs11797752	0.0580126	11
INPP5D	rs7583618	FAM71E2	rs7409446	0.0578879	80
FERMT2	rs78623109	CTD-2015H3.2	rs5002230	0.0578584	1
HLA-DRB5	rs141658384	FAM71E2	rs7409446	0.0578349	34
SORL1	rs67140863	MYO18B	22:26183256	0.0576567	4
FERMT2	rs78623109	KIAA0020	rs62534385	0.0574244	1
PTK2B	rs74614358	FAM71E2	rs7409446	0.0571105	108
FERMT2	rs78623109	BCORL1	rs12388749	0.056714	2
HLA-DRB5	rs148024059	PDZD8	10:119101909	0.0566114	4
PTK2B	rs377610860	RP11-298A8.2	rs5907739	0.0565009	3
FERMT2	rs78623109	RP4-799D16.1	rs71652350	0.056456	12
HLA-DRB5	rs73726202	TMEM100	17:53803074	0.0559538	10
CASS4	rs199744178	FAM71E2	rs7409446	0.0555891	23
FERMT2	rs78623109	IL20RB-AS1	3:136685211	0.055223	1
HLA-DRB1	rs111358198	CISD3	rs12452967	0.0551564	6
RIN3	rs7154465	FAM71E2	rs7409446	0.0550938	87
INPP5D	2:233994599	CNTN6	rs155399	0.0550713	4
SORL1	rs7104977	LPPR1	9:104001343	0.0548303	11
RIN3	rs139801732	YTHDC2	rs6893758	0.0547933	12
HLA-DRB1	rs111358198	SLC13A3	rs202389	0.0544929	7
HLA-DRB5	rs60378094	RP11-465I4.2	rs79406085	0.0543113	8
FERMT2	rs77026229	ZFHX4-AS1	rs28532385	0.0540396	9
CR1	rs2296160	FAM71E2	rs7409446	0.0538016	43
SLC24A4	rs10147792	FAM71E2	rs7409446	0.0536545	107
FERMT2	rs78623109	RP11-459E5.1	rs10111062	0.053576	12
ABCA7	rs3752234	SAMD12	rs9643127	0.053545	3
HLA-DRB1	rs115883159	DMD	rs55646021	0.0534203	14
RIN3	rs55968353	LPPR1	9:104001343	0.0533455	29
HLA-DRB1	rs41284728	FAM71E2	rs7409446	0.0531738	23
CLU	rs2070926	FBN3	rs371666740	0.0531343	10
FERMT2	rs77026229	RP11-655C2.3	11:58268329	0.0531318	1
HLA-DRB1	rs111659540	PDZD8	10:119101909	0.053022	1
NME8	rs6976216	FAM71E2	rs7409446	0.0529707	68
FERMT2	rs78623109	RP1-63G5.5	rs35407236	0.0529293	7
HLA-DRB5	6:32497709	MTMR1	rs16995747	0.052685	4
PICALM	rs35206999	FAM71E2	rs7409446	0.0524876	55
HLA-DRB1	rs111358198	AC133528.2	rs143076111	0.0523997	4
EPHA1	rs4283960	FAM71E2	rs7409446	0.0523772	10
HLA-DRB1	rs111358198	RP11-457K10.1	rs12629891	0.0523045	32
EPHA1	rs11762334	DNER	rs13027856	0.0522577	1
FERMT2	rs78623109	RP11-337C18.10	rs7517655	0.0521284	35
HLA-DRB1	rs111358198	DTYMK	rs111965544	0.0519647	3
FERMT2	rs78623109	RP11-276H19.2	rs1022995	0.0517166	1

Continued on next page

Continued from previous page

AD Gene		Other Gene			Total SNP pairs
Gene Name	rs ID or chr:pos	Gene Name	rs ID or chr:pos	r^2	for gene pair
FERMT2	rs78623109	COL4A6	rs5929103	0.0516701	1
DSG2	rs2704041	FAM71E2	rs7409446	0.0516222	24
HLA-DRB5	rs76939305	PDE4D	rs72765909	0.0515996	13
INPP5D	rs11684564	TMEM100	17:53803074	0.0515823	1
CASS4	rs67041321	INTS1	rs10241178	0.0515473	5
DSG2	rs2848681	FAM66E	rs145140607	0.0514365	34
INPP5D	2:233994599	RP11-268G12.3	rs6630361	0.0513256	21
INPP5D	2:233994599	PLXNB3	rs2266884	0.0512854	1
INPP5D	rs10199700	HSPD1P6	3:36822020	0.0512694	1
ABCA7	rs8109683	FAM71E2	rs7409446	0.0511257	8
INPP5D	2:233994599	RP11-317J19.1	rs11234716	0.0510802	8
DSG2	rs9945031	CASC18	12:106127457	0.0509782	18
FERMT2	rs55635639	RP11-124B13.1	rs193238269	0.0508982	15
HLA-DRB1	rs111358198	FAAH2	rs1048358	0.0508395	14
SORL1	rs58698151	INTS1	rs10241178	0.0507979	6
DSG2	rs7227984	MSH3	5:80044511	0.0507904	18
HLA-DRB5	rs113486815	RAB27A	rs28423052	0.0507894	1
FERMT2	rs78623109	RP1-154K9.2	rs182197422	0.0507179	1
MEF2C	rs35788185	FAM71E2	rs7409446	0.0506986	16
EPHA1	rs34824368	FAM71E2	rs7409446	0.0506494	2
SORL1	rs582446	FAM71E2	rs7409446	0.0506494	9
FERMT2	rs78623109	ADAM5	rs80022156	0.05054	7
EPHA1	rs2242601	MED13L	rs71442956	0.0505275	1
INPP5D	2:233994599	RP11-309M23.1	rs57527868	0.0504707	2
FERMT2	rs78623109	GNA12	rs148365357	0.05047	44
CASS4	rs6069753	LPPR1	9:104001343	0.0504167	14
FERMT2	rs78623109	E2F7	rs310791	0.050338	4
INPP5D	2:233994599	GRM5	rs308873	0.0501609	1
HLA-DRB1	rs17883271	RBM20	rs370447849	0.0500431	2
MS4A4E	rs80223119	ZNF275	rs1894352	0.049911	16
FERMT2	rs78623109	LINC01138	rs374755133	0.0498593	1
CELF1	rs61895102	FAM71E2	rs7409446	0.0498338	7
BIN1	rs4663096	FAM71E2	rs7409446	0.0498026	11
HLA-DRB1	rs111358198	ING5	rs35435997	0.04973	61
FERMT2	rs78623109	SKAP1	rs192894638	0.0497108	1
DSG2	rs2704051	LINC00969	rs34824969	0.0496771	37
FERMT2	rs78623109	METTL7B	rs6581077	0.0496693	5
HLA-DRB5	rs112634041	C2orf43	rs682961	0.0496473	9
FERMT2	rs78623109	CTC-254B4.1	rs150339186	0.0496294	1
HLA-DRB5	6:32492459	EIF3H	rs186798725	0.0496285	2
FERMT2	rs78623109	LINC00969	rs201104515	0.0495064	11
FERMT2	rs78623109	PRKAB2	rs10900321	0.0494792	15
CASS4	rs199744178	ZDHHC20	13:22022571	0.0494184	1
FERMT2	rs78623109	AMZ1	rs116929101	0.0494159	7
FERMT2	rs78623109	FBXO38	rs17638781	0.0493846	1
INPP5D	rs10195662	AC022816.2	17:14300732	0.0493707	2
FERMT2	rs77026229	TULP4	6:158814018	0.0493506	1
FERMT2	rs78623109	IKBIP	rs2289317	0.0492487	1
CASS4	rs386274	CERS6	2:169408885	0.0491953	2
INPP5D	2:233994599	GRIA3	rs5911575	0.0491564	1
FERMT2	rs78623109	LSAMP	rs28673788	0.0491519	1
DSG2	rs1460602	AP000275.65	rs1782987	0.0491269	6
FERMT2	rs78623109	DTX2P1	rs147939146	0.0491212	1
EPHA1	rs62472724	AL590867.1	6:153553842	0.0490696	6
INPP5D	rs10195662	INTS1	rs10241178	0.0490516	5
BIN1	rs13389409	SAMD12	rs9643127	0.0490159	6
FERMT2	rs78623109	ADAM3A	rs116911499	0.0488114	1
INPP5D	2:233994599	RP11-252A24.2	rs144611787	0.0487008	1
HLA-DRB5	rs187620826	TENM1	rs2050030	0.0487003	8
HLA-DRB5	rs114638398	CTD-3099C6.7	rs8110439	0.0486867	10
INPP5D	2:233994599	OTC	rs4537439	0.0486616	1
INPP5D	2:233994599	WDR72	rs11630682	0.0486151	8

Continued on next page

Continued from previous page

AD Gene		Other Gene			Total SNP pairs
Gene Name	rs ID or chr:pos	Gene Name	rs ID or chr:pos	r^2	for gene pair
NME8	rs55767998	INTS1	rs10241178	0.0486122	4
SLC24A4	rs8015378	RP11-124B13.1	rs193238269	0.0486019	78
FERMT2	rs78623109	ASTN2	rs10983474	0.0485498	3
SLC24A4	rs79363863	INTS1	rs10241178	0.0484547	5
INPP5D	2:233994599	LINC00632	rs5907635	0.0484032	1
DSG2	rs2848681	LINGO2	9:27989448	0.0483603	12
HLA-DRB5	6:32495860	PLXDC2	rs2478144	0.0483521	11
RIN3	rs75641131	TSGA10	rs11685765	0.0483369	4
FERMT2	rs78623109	CNBD1	rs13274049	0.0483232	2
INPP5D	2:233994599	UTP23	rs4464999	0.0482836	8
FERMT2	rs78623109	C17orf85	rs8068328	0.0482521	1
FERMT2	rs77026229	RP11-149I23.3	rs4933502	0.0482428	1
FERMT2	rs78623109	GRIA3	rs5956529	0.0481951	11
INPP5D	rs6761018	SAMD12	rs9643127	0.048106	5
SORL1	rs1699108	AC131180.1	2:131997258	0.0480866	15
HLA-DRB5	6:32497709	PCDH19	rs4828019	0.0480837	15
HLA-DRB5	rs139480376	GS1-433O24.1	rs4451489	0.048078	2
INPP5D	rs4335931	PKD1L2	rs78247837	0.0480311	3
FERMT2	rs78623109	ZNF888	rs10425203	0.047896	1
HLA-DRB5	rs113454243	ITIH6	rs17316505	0.0478843	1
FERMT2	rs78623109	AC011385.1	rs181919	0.0478686	1
HLA-DRB5	rs115356493	RP11-655C2.3	11:58268329	0.0478365	2
FERMT2	rs78623109	GJB1	rs184587106	0.0478272	1
HLA-DRB5	rs114967942	RPGR	rs5917557	0.0478196	1
INPP5D	2:233994599	PCOLCE-AS1	rs2686815	0.0478131	1
HLA-DRB5	rs112634041	RP11-162D9.3	rs57448945	0.0478064	24
HLA-DRB5	6:32497709	NDP	rs209762	0.0478021	3
CR1	rs144128028	TMEM100	17:53803074	0.047796	1
HLA-DRB5	rs199694873	CPN2	rs73892320	0.0477457	2
SORL1	rs67140863	KCNK2	rs200871376	0.0477261	2
PTK2B	rs976175	RBM20	rs370447849	0.0476879	23
FERMT2	rs77026229	RP11-362I1.1	rs71609453	0.047684	1
INPP5D	2:233994599	GLRA2	rs200277423	0.047619	1
SORL1	rs67140863	RP11-102J14.1	rs200719901	0.047522	3
FERMT2	rs78623109	GABRE	rs7341952	0.0475079	1
HLA-DRB5	rs113313722	ZNF100	19:21913252	0.0474928	5
EPHA1	rs11762334	RP4-576H24.4	20:1586443	0.0474876	4
EPHA1	rs3935067	INTS1	rs10241178	0.0474632	3
INPP5D	2:233994599	ME3	rs7123010	0.047445	13
INPP5D	rs10195662	SH3PXD2A	rs142308687	0.0474161	4
FERMT2	rs78623109	FTO	rs9937234	0.0473347	3
DSG2	rs7227984	MED16	rs2965290	0.0472685	14
HLA-DRB5	rs112634041	NMNAT2	rs10911301	0.0472477	20
FERMT2	rs78623109	RP11-326E22.1	rs7014635	0.0472381	2
FERMT2	rs78623109	AF196972.4	rs2977589	0.0471788	1
CELF1	rs113719483	APBA2	15:29261357	0.0471735	15
DSG2	rs73956136	LPPR1	9:104001343	0.0471585	6
FERMT2	rs78623109	RP11-141C7.3	rs139047772	0.0471562	2
HLA-DRB5	rs112634041	NREP	rs26047	0.0471412	3
EPHA1	rs4726618	RBM20	rs58752624	0.0471207	2
FERMT2	rs78623109	RP11-40F8.2	rs35661498	0.0471192	2
BIN1	rs60711884	RP11-124B13.1	rs193238269	0.0471094	5
DSG2	rs9945420	RBM20	rs370447849	0.0471018	5
HLA-DRB1	rs111358198	CSPG4P13	rs7162616	0.0470716	3
INPP5D	2:233994599	C12orf55	rs7962550	0.047067	18
INPP5D	2:233994599	KCNK1	rs7518174	0.0470276	2
INPP5D	2:233994599	EIF3H	rs4876680	0.0470196	6
FERMT2	rs78623109	SIGLEC14	rs885266	0.0469762	3
HLA-DRB5	rs114638398	DEFB125	rs892665	0.0469458	1
INPP5D	2:233994599	NYX	rs3013120	0.046928	7
HLA-DRB5	6:32492389	YTHDC2	rs6893758	0.0468987	13
APOE	rs769449	FAM71E2	rs7409446	0.0468325	1

Continued on next page

Continued from previous page

AD Gene		Other Gene			Total SNP pairs
Gene Name	rs ID or chr:pos	Gene Name	rs ID or chr:pos	r^2	for gene pair
SLC24A4	rs4904891	SPATA22	rs148500229	0.0467935	8
FERMT2	rs78623109	ZNF628	rs10423355	0.0467875	5
HLA-DRB5	rs112634041	CD4	rs56222055	0.0467496	6
PTK2B	rs10086912	RP11-124B13.1	rs193238269	0.0467428	13
CLU	rs9331950	INTS1	rs10241178	0.0467082	2
EPHA1	rs11762334	SLC29A4P1	rs79075337	0.0466343	1
HLA-DRB5	6:32493765	ABCA5	rs73998226	0.0466158	3
EPHA1	rs11767492	LPPR1	9:104001343	0.0465652	3
RIN3	rs4374096	RP4-576H24.4	20:1586443	0.0465622	3
FERMT2	rs3818453	YTHDC2	rs6893758	0.0464483	9
FERMT2	rs78623109	AC069513.4	rs374199070	0.0463969	1
INPP5D	2:233994599	RP11-856M7.6	rs78154263	0.0463958	1
INPP5D	2:233994599	GABRR2	rs2148174	0.0463464	3
INPP5D	2:233994599	CXXC1P1	rs12689158	0.0463198	1
CLU	rs9331896	FAM71E2	rs7409446	0.0463037	3
DSG2	rs1460602	HSPA4	5:132441799	0.0462841	6
FERMT2	rs3818453	RHPN2	rs115742337	0.0462553	10
RIN3	rs372072109	RP11-271F18.4	rs184141336	0.0462332	1
MS4A4E	rs11230188	FAM71E2	rs7409446	0.0461816	21

Concluded
